# Proposing the use of a physiotherapist navigator in an acute cancer care setting in Ontario: A nationwide survey using a modified Delphi approach

**DOI:** 10.1371/journal.pone.0355732

**Published:** 2026-08-07

**Authors:** Holly Edward, Danielle Lawrence, Alexander Grant, Alicia Page, Luciana Macedo, Sarah Wojkowski, Som D. Mukherjee, Jenna Smith-Turchyn

**Affiliations:** 1 School of Rehabilitation Science, McMaster University, Hamilton, Ontario, Canada; 2 Oncology Division, Canadian Physiotherapy Association, Ottawa, Ontario, Canada; 3 Oncology, McMaster University, Hamilton, Ontario, Canada; Touro University California College of Pharmacy, UNITED STATES OF AMERICA

## Abstract

**Objective:**

To engage health care providers in Canada to identify the roles, tasks, processes, assessments, and triaging decisions for a Physiotherapist (PT) Navigator role that will be implemented and evaluated in a future trial.

**Methods:**

A two-round, online, modified reactive Delphi study was used. Any licensed health care practitioner in Canada who works with individuals with or beyond cancer was eligible to participate. Round 1 identified qualitative feedback on role domains to guide role improvement. Qualitative feedback was examined in duplicate using content analysis. Round 2 identified quantitative feedback to gather numerical data to establish consensus. Quantitative survey feedback was analysed using descriptive statistics including mean and standard deviation. Items were added to the role description if identified by ≥10% of respondents, and 75% was the threshold to define consensus. Lastly, a consensus meeting including end users was held to consider priority items and utility of role translation into clinical practice.

**Results:**

Consensus on the PT Navigator role description was achieved following the two survey rounds (Round One; n = 34, Round Two; n = 18). Four domains were identified to define the role: process, assessment, triaging decisions, and duties and responsibilities. A figure visualizing the PT Navigator role was generated. Changes to the role after survey feedback included increasing appointment times with the PT Navigator, longer duration between follow-ups, patient self-referral, and additional objective testing. Consensus meeting feedback considered the variability in follow-up depending on different treatment timelines and an emphasis on promoting self-management strategies. Perceived usefulness of the role was high (mean 9.19/10; SD 2.12).

**Conclusions:**

This modified Delphi study identified key domains that would inform development of a comprehensive PT Navigator role working with cancer patients. The role offers a novel approach to promote routine assessment and early intervention, with the aim towards integrating PTs meaningfully within cancer care settings in Canada.

## Introduction

Many Canadians are diagnosed with cancer throughout their lifetime, with estimates as high as one in two [1]. Although there is an increase in cancer prevalence, cancer survival rates in Canada have similarly increased as a result from improvements in early detection and advancing medical treatments [2,3]. However, cancer and its associated treatments often result in numerous symptoms including pain, fatigue, weakness, and peripheral neuropathy which can affect multiple body systems impacting quality of life and overall functioning [4–6]. Extensive research has shown that physical activity and rehabilitation can reduce and prevent many of these negative symptoms amongst those living with and beyond cancer, [7,8] reduce disease recurrence [9], increase overall survival [9], and is recommended in various clinical practice guidelines [7,10–12]. Despite the clear and consistent published literature demonstrating the benefits of physical activity in cancer patients, only about 30% of cancer survivors in Canada have access to rehabilitation services [13].

Physiotherapists (PTs) are regulated health care professionals and experts in the field of rehabilitation and health promotion in Canada [14]. Current provincial [10] and international [11,12] oncology clinical practice guidelines recommend a multi-disciplinary approach within the cancer care team that includes PT involvement during and after cancer treatment. Many countries, including Canada, do not include or provide PT services within their cancer centres [15], which leaves individuals with cancer on their own to find rehabilitation professionals in the community and cover associated costs. In addition, many Canadians living with or beyond cancer do not seek out rehabilitation, despite the clear benefits [16,17]. There is a pronounced gap in rehabilitation services for Canadians living with cancer with the most challenges related to accessing care to manage long-term impairments [18].

Navigation in health care has been used as a care model to meet the needs of health systems worldwide [19]. The first navigational program was created in 1990, as a response to reducing the barriers and the time between the point of cancer suspicion to diagnosis and treatment in the United States of America [20]. Since then, the scope of patient navigation has expanded across all stages of cancer care from prevention, early detection/diagnosis, treatment, survivorship and end of life care [20]. The introduction of PTs in navigational roles has been increasingly studied and implemented in cancer centres over the past ten years [21]. Navigation across the cancer care continuum has commonly been performed by nurses who provide patients with education, monitor medication/treatment adherence, and assist with treatment initiation [22,23]. A recent scoping review explored PT Navigator roles currently used in cancer care internationally and identified that PT Navigators often engage in screening/triaging functional changes, provide referrals to other health care disciplines, implement rehabilitation interventions, identify treatment barriers, and support goal setting [21]. Almost all of the thirteen references included in the review (n = 11; 84.6%) were from the United States of America, with the remaining references from Canada and Mexico [21]. Exploring the integration of PTs within these roles in Canada can facilitate early identification of physical and functional impairments through assessment at various stages of cancer treatment (i.e., a Prospective Surveillance Model [24]). This role may offer an efficient approach to increase access to care and minimize immediate and long-term morbidity secondary to cancer and its treatments. The objective of this study is to engage health care providers in Canada to identify the duties, processes, assessments, and triaging decisions of a PT Navigator role prior to implementation in a future trial.

### Research question

1) What are the duties, processes, assessments, and triaging decisions of a PT Navigator in an acute cancer care setting (i.e., a setting administering cancer treatments to individuals living with cancer) in Canada?

## Materials and methods

### Study design

This study employed an online, modified reactive Delphi approach [25–27]. This approach utilizes a structured process that gains the views of experts to establish a consensus over a succession of survey rounds [25,26]. This study was reactive as participants were asked to rate a pre-developed PT Navigator role description, while posing open-ended questions to seek feedback/suggestions on how to modify such role [25]. This technique has been commonly used to develop quality assessment indicators [25,27–29]. The authors decided a priori to limit data collection to two rounds as recommended in health care research while aiming to avoid survey fatigue [30]. A consensus meeting with end-users was scheduled following completion of both survey rounds to review the changes made to the role description and to consider the practicality of role translation into clinical practice. Also, to strengthen the reporting of our results, this study followed the Delphi studies in social and health sciences–recommendations for an interdisciplinary standardized reporting (DELPHISTAR) guidelines [31]. There is no published protocol for this study. The Hamilton Integrated Research Ethics Board in Hamilton, Ontario, approved this study (ID: 18218). Informed consent was obtained electronically from all participants prior to each survey round.

### Participants

To be included in the study and complete the online surveys, participants must have been physiotherapists or other health professionals (e.g., oncologists, nurses, allied health) practicing in Canada who work with individuals living with and/or beyond cancer in any setting (e.g., hospital, private practice). Participants also needed to be able to understand and write in English. Recruitment emails were distributed through the Canadian Physiotherapy Association’s (CPA’s) Oncology Division monthly member e-blasts, and to physicians at the local cancer centre in Hamilton, Ontario. Study advertisements were also shared on CPA’s Oncology Division’s social media pages (i.e., Facebook, Instagram).

### Survey development

The development of the first-round survey and PT Navigator role description was informed by results of a scoping review that explored the current literature on the use of PT Navigators in acute cancer care settings [21]. Data was organized by the lead author (HE) into four domains based on concepts described in the review and guided by a professional navigation framework [32]: process, assessment, triaging decisions, and main duties and responsibilities. The initial survey design, instructions, role description, and questions underwent multiple rounds of review, revision, and pilot testing by members of the study team, which included four physiotherapists (HE, JST, SW, LM), and a medical oncologist (SM). Basic demographic data was collected at both the first and second round of surveys regarding each participant’s profession, area of practice, and province or territory of practice.

### Sample size

As a modified Delphi study, our sample size was determined relative to group dynamics in achieving consensus and aimed to recruit participants who represent various disciplines rather than achieving statistical power [33,34]. In Delphi studies, a minimum of 10–18 respondents is recommended [33,35]. However, Delphi studies can have variable attrition rates ranging from 0–92% [36]. To account for a 20% drop out rate between the two surveys, we aimed to recruit at least 23 participants for the first survey, while considering the more participants included would lead to a greater range of opinions using a purposive sampling strategy.

### Procedure

The project included two rounds of surveys and a final consensus meeting. Each round was delivered online using LimeSurvey (LimeSurvey GmbH, Hamburg, Germany). See S1 File for a copy of the first and second survey that was administered. Anonymity was maintained during all survey rounds and the consensus meeting through de-identification of responses.

**Fig 1:**
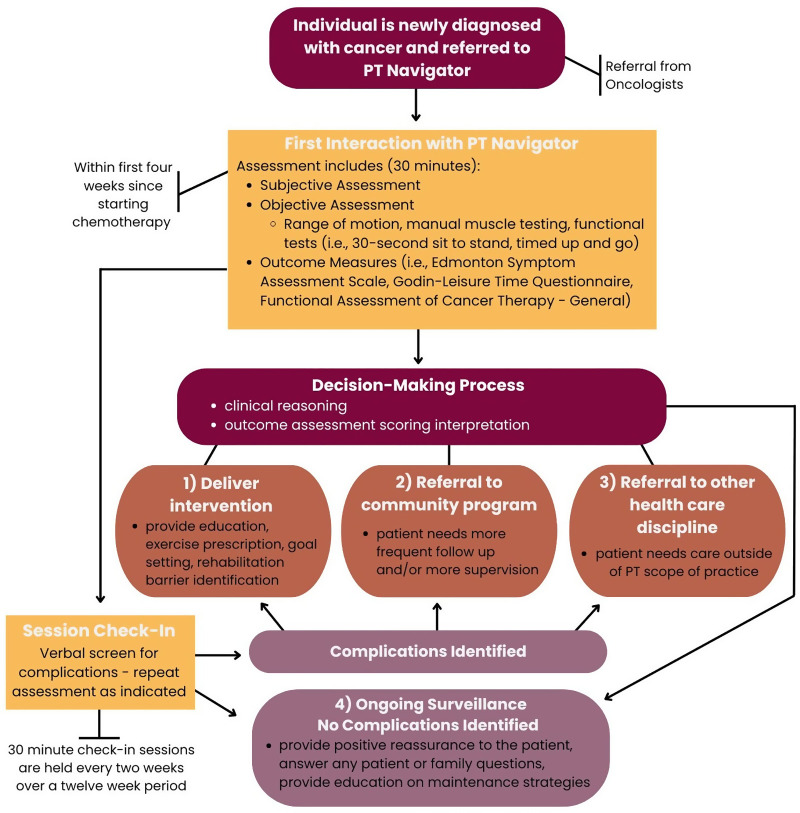
Role process figure design: round one.

**Fig 2:**
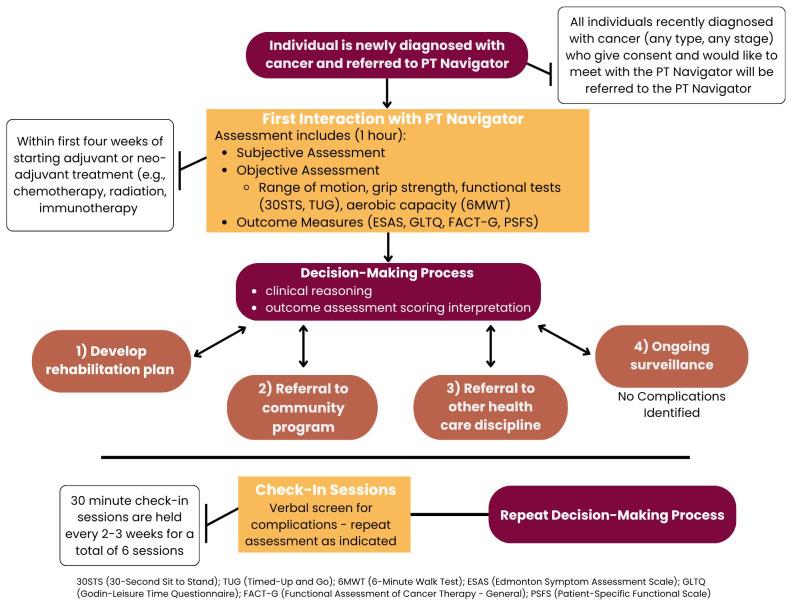
Role process figure design: final after consensus meeting.

Round 1. The first survey was open for 10 weeks (Jan 8 to Mar 19, 2025) and took approximately 15 minutes to complete. The questions in the first survey included qualitative open-ended responses relating to the PT Navigator role across four domains (process, assessment, triaging decisions, duties and responsibilities, and a figure visualizing the PT Navigator role process). Qualitative questions asked participants to comment on what they liked and what was missing/what could be improved related to each domain. Two additional quantitative questions were used at the end of the survey. The first quantitative question asked participants to score the perceived usefulness of the role on a scale of 1–10 (1 – not useful at all to 10 – very useful) with an optional text box to comment on selected score. The second quantitative question asked participants to score the clarity of the figure visualizing the PT Navigator process (see Fig 1 and 2) on a scale of 1–5 (1 – very unclear and cannot understand to 5 – clear and understandable) with an optional text box to comment on selected score. Following results of the first survey, the role was updated and used to create the survey in Round 2.

Round 2. The second survey was activated two weeks following the closure of the first survey, was open for two weeks (Apr 2 to Apr 16, 2025) and followed the same structure but included rating and agreement selections to quantify role accord. The second survey took approximately 10 minutes to complete. Only participants who completed the first survey were eligible to complete the second survey. In the second survey, participants were asked to evaluate the updated PT Navigator role description. The questions in the second survey were designed with a quantitative focus to gather numerical data with the aim of establishing consensus. For each of the four domains and figure design, participants were asked to rate the importance of each criterion using a 7-point Likert scale (1 – strongly disagree to 7 – strongly agree) [37]. An optional text box was added after each criterion to allow participants who selected neutral or below to provide comments on the individual item.

Consensus Meeting. After both surveys were completed and updates were made to the role description, an online consensus meeting was held to review results and finalize the PT Navigator role description. The final consensus meeting was held and recorded over Zoom (Zoom Communications Inc, California, United States) in June 2025. The end users in attendance represented a variety of professions (e.g., physiotherapists, researchers, a medical oncologist, and a patient advocate). These end users were determined a priori based on their expertise in oncology, rehabilitation, patient experience, and health care models and services including triaging. During the meeting, all attendees were asked to consider each role domain and the changes that were made based on participant feedback from each of the survey rounds. Attendees were also asked to acknowledge whether changes were made appropriately and consider the practical implications of testing a role within a local cancer centre. Individual items of the role description established in the second survey were examined and included in the final description if consensus was established amongst meeting participants.

### Data analysis

Data from both online surveys were analyzed using descriptive statistics (i.e., means, standard deviations, frequencies, percentages) where appropriate. Open ended survey questions were analyzed using content analysis performed in duplicate (HE and DL) to inform recommendations based on feedback from participants. Discrepancies in coding were resolved through discussion between reviewers. Any new items to the role description were added to Round 2 if suggested by more than 10% of participants [38,39]. Seventy five percent was the median threshold to define consensus [40,41]. Consensus amongst each survey domain in Round 2 was established if the item scored a mean of 5.25/7 (i.e., 75%). Results from the analyses and consensus round were used to update the role description that will be trialed in a future pilot RCT.

## Results

### Participants

For Round 1, a total of 34 participants completed the survey. For Round 2, 19 participants from Round 1 consented to being contacted to participate in the second survey (n = 19/34; 55.9%) and 18 completed Round 2 (n = 18/19; 94.7%). Participants from both surveys represented a diverse range of health professionals from various provinces/territories across Canada. See Table 1 for characteristics of survey respondents. The consensus meeting included seven end-users with various backgrounds such as physiotherapists (n = 6/7; 85.7%), researchers (n = 3; 42.9%), a medical oncologist (n = 1; 14.3%), and a patient advocate (n = 1; 14.3%). Survey results have been included as supporting information, see data in S2 and S3 Files.

**Table 1:** Characteristics of Participants.

	*Round 1* (n = 34)	*Round 2* (n = 18)
***Type of Health Professional***	***n (%)***	***n (%)***
Physiotherapist	24 (70.6)	14 (77.7)
Oncologist	4 (11.8)	1 (5.6)
Resident Physician	2 (5.9)	0 (0)
Nurse	2 (5.9)	2 (11.1)
Physiotherapy Resident	1 (2.9)	0 (0)
Massage Therapist	1 (2.9)	1 (5.6)
**Area of Practice**		
Private practice	10 (29.4)	7 (38.9)
In-patient hospital	9 (26.5)	3 (16.7)
Multi-setting (i.e., out-patient and in-patient hospital)	5 (14.7)	1 (5.6)
Out-patient hospital	3 (8.8)	2 (11.1)
Research	2 (5.9)	2 (11.1)
Community	2 (5.9)	1 (5.6)
Multi-setting (i.e., out-patient, in-patient hospital, and research)	1 (2.9)	1 (5.6)
Multi-setting (i.e., private practice and research)	1 (2.9)	1 (5.6)
Multi-setting (i.e., research and administrative)	1 (2.9)	0 (0)
**Location of Practice in Canada**		
Ontario	22 (64.7)	9 (50)
British Columbia	5 (14.7)	2 (11.1)
Alberta	4 (11.8)	6 (33.3)
Manitoba	1 (2.9)	1 (5.6)
Newfoundland and Labrador	1 (2.9)	0 (0)
Yukon Territory	1 (2.9)	0 (0)

### Round 1

Qualitative responses identified seven themes meeting the threshold for inclusion of new items in the Round 2 survey (see Table 2). These were distributed across process (n = 3/7; 42.9%), assessment (n = 2/7; 28.6%), triaging decisions (n = 1/7; 14.3%), and the role process figure (n = 1/7; 14.3%). The most frequently cited suggestion was the need for more objective testing within the assessment domain (n = 10/34; 29.4%). Quantitatively, the role demonstrated high perceived usefulness (mean 9.19/10, SD 2.12) and high figure clarity (mean 4.39/5, SD 0.96) (see Fig 1).

**Table 2:** Round One Survey Results: Thematic Analysis of Feedback for Role Improvement (n = 34).

Themes	Count (n; %)
**Process**	
**Missing individuals who receive other treatments/schedules** **Assessment is too short** **Need for additional referral opportunities (e.g., self-referral)** Consideration for out-patient vs in-patient settings Consider offering virtual follow-ups	**n=6; 17.6%** **n=6; 17.6%** **n=5; 14.7%** n=2; 5.9% n=2; 5.9%
**Assessment**	
**Need for more objective testing** **Include lymphedema assessment** Need to include aerobic testing Tailored assessment per patient vs standard set of outcome measures Include a peripheral neuropathy assessment Consider spiritual history Include a frailty assessment	**n=10; 29.4%** **n=6; 17.6%** n=3; 8.8% n=3; 8.8% n=2; 5.9% n=2; 5.9% n=1; 2.9%
**Triaging Decisions**	
**MMT/goniometry too subjective** Use of clinical reasoning vs normative data to guide triaging Consider financial support for community referrals	**n=4; 11.8%** n=2; 5.9% n=1; 2.9%
**Duties and Responsibilities**	
Reactionary vs proactive approach to triaging Facilitating exercises classes for patients Bridge the gap between inpatient-outpatient services Supervise physiotherapy students Maximize scope through medical directives (e.g., order a CBC, referral to physiatrist)	n=1; 2.9% n=1; 2.9% n=1; 2.9% n=1; 2.9% n=1; 2.9%
**Figure Clarity**	
**Busy figure (reduce arrows, colours)**	**n=4; 11.8%**

*MMT; Manual Muscle Testing, CBC; Complete Blood Count

### Round 2

In Round 2, all items within the PT Navigator role achieved consensus (≥75% agreement), and no further modifications were required (see Table 3). No comments or suggestions were reported by more than one participant for any item.

**Table 3:** Round Two Survey Results (n = 18).

Domain	Rating (mean; SD)
**Process**	
The process is clear and easy to follow I am satisfied with the process of the PT Navigator	6.39; 0.61 5.83; 1.10
**Assessment**	
The assessment is clear and easy to follow I am satisfied with the assessment of the PT Navigator	6.53; 0.51 6.29; 0.77
**Triaging Decisions**	
The triaging decisions are clear and easy to follow I am satisfied with the triaging decisions of the PT Navigator	6.24; 0.97 6.12; 1.05
**Duties and Responsibilities**	
The duties and responsibilities of the PT Navigator are clear and easy to follow I am satisfied with the duties and responsibilities of the PT Navigator	6.25; 1.06 6.5; 0.63
**Figure**	
The updated figure is clear and easy to follow I am satisfied with the updated figure	6.44; 0.73 6.33; 0.73

### Consensus meeting

Attendees agreed with all changes made within the survey rounds but highlighted some issues that would need to be addressed when testing the role in a research setting. This included considerations for various follow-up times after cancer surgery (e.g., a patient who received cancer treatment surgery may need a different follow-up time interval than a patient coming to the cancer centre to receive chemotherapy on two-to-three-week cycles). Additionally, participants agreed the PT should focus on promoting self-management education and referring rather than providing hands-on treatment within the acute cancer care setting. As a result, the role document was updated so the process domain was reflective of this emphasis of care navigation rather than providing timely/advanced physiotherapy treatment (e.g., manual therapy such as manual lymphatic drainage, joint mobilizations). To better represent this within the triaging decisions role domain, the heading ‘Deliver Physiotherapy Intervention’ was updated to ‘Develop Rehabilitation Plan’. The heading and treatment follow up considerations was also updated within the figure visualizing role process, and some additional boxes were added around figure text to increase overall clarity, see Fig 2. The final poll demonstrated universal agreement by all meeting attendees (n = 7/7) of the PT Navigator role with approval towards testing the identified role in an upcoming pilot RCT. See S4 File for the finalized PT Navigator role description.

## Discussion

This study engaged health care professionals across Canada to develop a novel PT Navigator role description to implement in a cancer centre in Canada. Participants reached consensus on the PT Navigator role domains proposed in survey rounds and in consensus meetings. Changes to the role domains based on survey responses centred around role process (i.e., longer appointment times with the PT Navigator, increased duration between follow-ups, and the ability for patients to self-refer), assessment (i.e., additional objective testing), and triaging decisions (i.e., decisions based on more objective measures rather than subjective outcomes such as manual muscle testing). End users in the consensus meeting reviewed the changes made to the role description and considered factors that would influence translation of the role in research settings such as the variability in follow-up depending on different treatments timelines (e.g., chemotherapy compared to surgery) and an emphasis on promoting self-management strategies. Strong agreement and qualitative feedback throughout all domains demonstrate positive support and reinforcement to the integration of a PT within acute cancer care settings.

The item that received the most feedback on adding to the role proposal was the need for more objective testing within the assessment domain. This finding highlights important considerations when determining which assessment methods to use to identify early impairments of cancer and its treatments, and that patient-rated measures alone cannot sufficiently capture the degree of loss of function [42].However, clinicians and researchers will also need to consider limitations of objective testing, such as singular outcome measures that assesses only one domain of function (e.g., six-minute walk test measures aerobic capacity, handgrip strength measures strength) [42]; and that certain measures might be difficult to administer in clinic due to equipment and space requirements and the time require to administer [43]. It will be critical for clinicians and researchers to consider assessing various health-related outcomes using both subjective and objective-based measures considering the different types of cancer and the unique demands that individuals living with cancer will face. Further, future evaluation of the PT Navigator role should consider both patient-reported outcomes (e.g., function, self-management, and quality of life) and service-level indicators (e.g., health care utilisation and referral patterns), alongside feasibility and acceptability measures.

An important consideration, many symptoms that cancer patients experience are multi-dimensional which can make diagnosis of their condition and supportive treatment challenging [44,45]. It is estimated that individuals living with cancer may experience numerous concurring symptoms during treatment (e.g., median ranges from 6–29 symptoms) [46,47]. The PT Navigator will need to consider a symptom network approach acknowledging the dynamic relationship of symptom clusters (e.g., two or more symptoms that are associated to one another and occur together) to design effective management strategies as well as to triage effectively [48–50]. As highlighted by participant responses, and emphasized throughout the role description, to triage participants to the health care services, education, or rehabilitation programs they require accurately and effectively, the PT Navigator will need to tailor their assessment to ensure patient-centered care while including many outcome measures to guide their recommendations. In addition, the PT Navigator working with cancer patients should have at least a basic understanding of the major types of cancer treatments and their side effects including surgery, radiation, chemotherapy, immunotherapy, targeted therapies and anti-hormonal therapy to allow them to better understand the interactions between their treatment, symptoms and physical function.

Also as highlighted within a survey response and to align with a prospective surveillance approach [24], the PT Navigator will need to consider a reactionary vs proactive approach to identifying and managing cancer and treatment-related side effects. A recently published survey study [51] explored patient-related experiences regarding side effects during breast cancer treatment. A significant number of participants (14.4% to 46.8% across different side effects) reported receiving information on side effects only after they were first experiencing the side effect [51]. Further, approximately 50% of the discussions regarding side effects were initiated by the patients themselves, suggesting a reactive approach by health care professionals [51]. The lack of symptom-focused communication can result in poor care coordination, insufficient referrals to other health care services, affect treatment adherence, and affect patient outcomes such as quality of life [51–54]. The PT Navigator role emphasizes symptom identification and early management through routine monitoring early into cancer treatment. However, to further distinguish between reactive and proactive symptom monitoring and triaging, the PT Navigator should use clinical reasoning to anticipate patient needs and tailor assessment measures according to the patient's presentation, symptom burden, and treatment stage, rather than relying solely on outcome assessment scores [55]. After any identification of symptoms or suspicion of symptom progression, it will be important for the PT Navigator to communicate with the oncology team (i.e., oncologists, nurses) so that the team can act to facilitate a proactive approach to care (e.g., prescribe medication for nausea, pain, assess for infection). The data collected from the planned pilot trial and further research looking at predictive modelling will be beneficial to further inform proactive triaging approaches.

While support for the PT Navigator role was evident within participant responses, it would be interesting to consider expanding the duties and responsibilities of the Navigator and optimizing how health services are accessed as suggested by survey participants. For example, maximizing the scope of practice of the PT through medical directives including ordering a complete blood count to understand whether a low hemoglobin, or anemia, could be contributing to a patients’ level of fatigue, symptoms of weakness or declining function. In addition, a PT navigator role could be more effective if there was an expanded ability to direct referral to other medical specialists such as physiatrists. This type of advanced practice has been used by PTs globally and there is evidence to support effectiveness with increasing accessibility of care, reducing wait times, and assisting with workforce shortages [56–58]. Currently in Ontario, medical directives are written orders by physician(s) to other licensed health professionals that allow them to carry out treatments or procedures that are specified under that directive, provided certain conditions are met [59]. With a medical directive in place, the PT Navigator would be permitted to perform such tasks that are currently outside of their scope of practice [60], which would further assist in removing barriers to care and enabling patients to access the treatment they need. However, given the administrative load and cost of implementing and maintaining medical directives, this form of delegation may not be sustainable in the long term [61]. It will be important for future research into navigational and triage roles to consider the local scope of practice of PTs and further explore ways to involve PTs as active members of cancer care teams.

### Limitations

The findings of this study need to be interpreted within the context of its limitations. First, there is a likelihood of personal, professional, and sampling bias towards support for this role, as most participants were practicing PTs recruited through physiotherapy networks. Consequently, the consensus findings may disproportionately reflect physiotherapy perspectives regarding the role, responsibilities, and competencies required for this position. Given that this role is intended to function within a multidisciplinary oncology care environment, the limited representation of other health care professions may affect the transferability and generalizability of the findings across disciplines. Further, only one individual living beyond cancer participated in the consensus panel, alongside health care professionals only, meaning that patient perspectives were limited in the development process. This is an important limitation given the intention for the model to support patient-centred care. In addition, perspectives from other key stakeholders, including additional individuals living with cancer, hospital managers, and executive leadership, were not captured. Also, only 55.9% of respondents from the first survey consented to be contacted for the second round which limited participant responses for the second survey. With this high attrition rate, it is important to acknowledge the possibility of non-response bias, whereby the views of participants who did not complete subsequent rounds may differ systematically from those who remained. Although consensus was achieved according to our predefined criteria, within the final Delphi sample participant attrition and the limited diversity of the survey respondents may have influenced the range of perspectives captured.

## Conclusion

This study leveraged modified Delphi methodology to inform the development of a PT Navigator role representing an important step towards identifying strategies to integrate PTs within acute cancer care settings in Canada. The role aims to promote routine assessment and early intervention to improve the function and quality of life of individuals living with and beyond cancer. Results of this study aim to maximize the success and feasibility of the role when trialed in an upcoming pilot RCT. Additionally, future research will be needed to establish cost-effectiveness to support funding opportunities for such positions within cancer care teams.

## Supporting information

S1 File Copy of surveys. (DOCX)

S2 File Round one survey results. (XLSX)

S3 File Round three survey results. (XLSX)

S4 File PT navigator role description. (DOCX)
