# Supplementary material for: Proposing the use of a physiotherapist navigator in an acute cancer care setting in Ontario: A nationwide survey using a modified Delphi approach
Source: PLoS One. 2026 Aug 7;21(8):e0355732. doi: 10.1371/journal.pone.0355732 (PMC13450761; doi:10.1371/journal.pone.0355732)
Supplement: Supplementary file 1 [file pone.0355732.s001.docx]

**Appendix 1. Round One and Two Survey**

***Round One***

**A Novel Role Proposal for the Use of a Physiotherapist Navigator in an Acute Cancer Care Setting in Ontario: A Nationwide Survey**

Thank you for your interest in participating in this survey! This survey is administered by Holly Edward, PT, PhD Candidate and Dr. Jenna Smith-Turchyn, PT, PhD from McMaster University. The purpose of this survey is to gain feedback on a new role proposal (a Physiotherapy Navigator) for use in an acute cancer care setting in Ontario (i.e., a setting administering cancer treatments for individuals with a current diagnosis of cancer) from physiotherapists and other health professionals in Canada.

**Please click the link below to download and read the accompanying letter of information.**

This letter contains more details regarding the survey and the researcher’s study, information related to confidentiality, anonymity, withdrawal procedures and more. This survey is part of a study that has been approved by the Hamilton Integrated Research Ethics Board (HiREB). The HiREB protocol number associated with this survey is #18218.

**Letter of Information:** [/limesurvey/upload/surveys/479331/files/Consent%20Form-Survey-Dec%205%20-%20Clean.docx](https://surveys.mcmaster.ca/limesurvey/upload/surveys/479331/files/Consent%20Form-Survey-Dec%205%20-%20Clean.docx)

Consent to Participate

Having read the above, I understand that by clicking the “Yes” button below, I agree to take part in this study under the terms and conditions outlined in the accompanied letter of information.

Please choose **only one** of the following:

- Yes, I agree to participate
- No, I do not agree to participate

Demographic Information

Please select the option that best describes you:

 Choose one of the following answers

 If you choose 'Other' please also specify your choice in the accompanying text field.

Please choose **only one** of the following:

- PT
- PT Resident
- Oncologist
- Nurse
- Other:

What area of practice do you work with individuals living with and/or beyond cancer?

 Check all that apply

Please choose **all** that apply:

- Out-patient hospital
- In-patient hospital
- Private practice
- Research
- Other:

What province or territory do you practice in?

 Check all that apply

Please choose **all** that apply:

- Alberta
- British Columbia
- Manitoba
- New Brunswick
- Newfoundland and Labrador
- Nova Scotia
- Ontario
- Prince Edward Island
- Quebec
- Saskatchewan
- Northwest Territories
- Nunavut
- Yukon

PT Navigator Role

**In the next few questions, you will be asked about feedback on the proposed role of a Physiotherapy (PT) Navigator in acute cancer care settings (i.e., a setting administering cancer treatments to individuals with a current diagnosis of cancer) to be trialed in an upcoming pilot randomized control trial. The purpose of the role is to reduce cancer-related morbidity using prospective monitoring and surveillance to optimize health, physical function and quality of life.**

*Process*

- Oncologists will refer individuals recently diagnosed with cancer (any type, any stage) to the PT Navigator
- Within the first month of starting chemotherapy treatment, the PT Navigator will perform a 30-minute subjective and objective assessment and administer evidence-based self-reported outcome measures validated for use in oncological populations
- Following the assessment, the PT Navigator will use their clinical reasoning and outcome measure scoring interpretations detailed in a future question to guide decision making on next steps
- The PT Navigator will follow up with the patient every two weeks for twelve weeks (for approximately 30-minutes) to check-in on their status
- To increase accessibility, sessions with the PT Navigator will be scheduled for when the patient is already coming to the cancer centre for a scheduled chemotherapy treatment and/or appointment with their oncologist
- The PT Navigator will be notified that the patient has arrived following their check-in with the oncology care team and will receive the patient from the waiting room
- Check-in appointments will include a verbal screen for complications and repeat assessments as indicated

What do you like about the process?

Please write your answer here:

What is missing? How can the process be improved?

Please write your answer here:

*Assessment*

- Subjective: will include an assessment of current symptoms, history of present illness, medical history, social history, physical activity history, review of medications, and other pertinent information
- Objective: will include tests such as range of motion using goniometry, muscle strength testing (MMT), and functional tests (30-second sit-to-stand, timed up and go)
- Outcome Measures:
  - Edmonton Symptom Assessment Scale
  - Godin-Leisure Time Questionnaire
  - Functional Assessment of Cancer Therapy – General (FACT-G)

What do you like about the assessment?

Please write your answer here:

What is missing? How can the assessment be improved?

Please write your answer here:

*Triaging Decisions*

- The PT Navigator will move forward with one of the following steps based on their assessment:
  - 1) Deliver a physiotherapy intervention (i.e., provide education, exercise prescription, goal setting, rehabilitation barrier identification)
  - 2) Refer the individual to a community program (i.e., the patient needs more frequent follow-up and/or more supervision with rehabilitation services)
  - 3) Refer to another health care discipline (i.e., the patient needs care outside of the PT scope of practice – for example, to discuss changes in weight and appetite with a dietician)
  - 4) Continue with ongoing surveillance (i.e., no complications of chemotherapy treatment identified – continue to provide positive reassurance, answer questions, and provide education on maintenance strategies)

Below is an accompanying guide created to support triaging based on outcome-assessment scores:

 What do you like about how triaging decisions will be made?

Please write your answer here:

What is missing? How can the decision-making process be improved?

Please write your answer here:

*Duties and Responsibilities*

The following picture contains the main duties and responsibilities proposed for the PT Navigator.

How useful on a scale of 1-10 is a PT Navigator role like this?

Please choose the appropriate response for each item:

Please explain your score provided above.

Please write your answer here:

Is there any other duty or responsibility that you think the PT Navigator should be responsible for? Is there anything you disagree with from the list above?

Please write your answer here:

*Figure*

The following is a figure visualizing the PT Navigator role process.

How would you rate the clarity of this figure visualizing the PT Navigator role process? (1 very unclear and cannot understand - 5 clear and understandable)

Please choose the appropriate response for each item:

Please explain your score above.

Thank you

**Thank you for taking the time to complete this survey. Results will be analyzed within one week of the survey closing and an updated role description will be available for a second round of feedback delivered in the same survey format.**

Are you available and/or interested in participating in the second survey? Note: only those who consent to being contacted about the second survey will be able to participate. The second survey will be available one week after this first survey has closed.

Please choose **only one** of the following:

- Yes
- No

If yes: please provide your email address so we can contact you:

Please write your answer here:

Lastly, would you like to provide your email address so we can contact you to share study results?

Please write your answer here:

Thank you for taking this survey. Your answers are a valuable part of this research.

**Round Two**

Survey Two: A Novel Role Proposal for the Use of a Physiotherapist Navigator in an Acute Cancer Care Setting in Ontario: A Nationwide Survey

Welcome and Consent

Thank you for your agreeing to take part in our **second survey** exploring feedback on a new role proposal (a Physiotherapy Navigator). The PT Navigator role description was modified based on feedback from responses of the first survey.

**Changes were made and new items added to the role description if suggested by more than 10% of participants.**

As a reminder, please see below for information from the first survey around consent to participate.

This survey is administered by Holly Edward, PT, PhD Candidate and Dr. Jenna Smith-Turchyn, PT, PhD from McMaster University. The purpose of this survey is to gain feedback on a new role proposal (a Physiotherapy Navigator) for use in an acute cancer care setting in Ontario (i.e., a setting administering cancer treatments for individuals with a current diagnosis of cancer) from physiotherapists and other health professionals in Canada.

Please click the link below to download and read the accompanying letter of information. This letter contains more details regarding the survey and the researcher’s study, information related to confidentiality, anonymity, withdrawal procedures and more. This survey is part of a study that has been approved by the Hamilton Integrated Research Ethics Board (HiREB). The HiREB protocol number associated with this survey is #18218.

Letter of Information: [/limesurvey/upload/surveys/479331/files/Consent%20Form-Survey-Dec%205%20-%20Clean.docx](https://surveys.mcmaster.ca/limesurvey/upload/surveys/479331/files/Consent%20Form-Survey-Dec%205%20-%20Clean.docx)

Consent to Participate

Having read the above, I understand that by clicking the “Yes” button below, I agree to take part in this study under the terms and conditions outlined in the accompanied letter of information.

Please choose **only one** of the following:

- Yes
- No

Demographic Information

Please select the option that best describes you:

Please choose **only one** of the following:

- PT
- PT Resident
- Oncologist
- Nurse
- Other:

What area of practice do you work with individuals living with and/or beyond cancer?

Please choose **all** that apply:

- Out-patient hospital
- In-patient hospital
- Private Practice
- Research
- Other:

What province or territory do you practice in?

Please choose **all** that apply:

- Alberta
- British Columbia
- Manitoba
- New Brunswick
- Newfoundland and Labrador
- Nova Scotia
- Ontario
- Prince Edward Island
- Quebec
- Saskatchewan
- Northwest Territories
- Nunavut
- Yukon

PT Navigator Role

In the next few questions, you will be asked for feedback on the **updated** PT Navigator role proposal for implementation in Canada. The purpose of the role is to reduce cancer-related morbidity using prospective monitoring and surveillance to optimize health, physical function and quality of life.

*Changes/additions to the role description can be identified by the* ***bold*** *lettering.*

*Process*

- **All** individuals recently diagnosed with cancer (any type, any stage) **who give consent and would like to meet with the PT Navigator** will be referred to the PT Navigator
- Within the first month of starting treatment **(e.g., chemotherapy, radiation, immunotherapy)** **or prior to cancer treatment surgery**, the PT Navigator will perform a subjective and objective assessment **(lasting approximately 1 hour)** and administer evidence-based self-reported outcome measures validated for use in oncological populations
- Following the assessment, the PT Navigator will use their clinical reasoning and outcome measure scoring interpretations to guide decision making on next steps
- The PT Navigator will follow up with the patient every **two to three** weeks **for a total of** **six sessions**
  - To increase accessibility, sessions with the PT Navigator will be scheduled for when the patient is already coming to the cancer centre for a scheduled treatment and/or appointment with their oncologist
- The PT Navigator will be notified that the patient has arrived following their check-in with the oncology care team and will receive the patient from the waiting room
- Check-in appointments (approximately 30-minutes long) will include a verbal screen for complications, repeat assessments as indicated, and **consider triaging decisions based on their findings and clinical reasoning**

Please rate the following statement: **The process is clear and easy to follow.**

Please choose the appropriate response for each item:

| **Strongly Disagree** | **Disagree** | **Somewhat Disagree** | **Neutral** | **Somewhat Agree** | **Agree** | **Strongly Agree** |
| --- | --- | --- | --- | --- | --- | --- |

If you rated neutral or below, please explain your selection provided above.

Please write your answer here:

Please rate the following statement: **I am satisfied with the process of the PT Navigator.**

Please choose the appropriate response for each item:

| **Strongly Disagree** | **Disagree** | **Somewhat Disagree** | **Neutral** | **Somewhat Agree** | **Agree** | **Strongly Agree** |
| --- | --- | --- | --- | --- | --- | --- |

If you rated neutral or below, please explain your selection provided above.

Please write your answer here:

*Assessment*

- Subjective: will include an assessment of current symptoms, history of present illness, medical history, social history, physical activity history, review of medications, and other pertinent information
- Objective: will include tests such as range of motion using goniometry, **grip strength,** functional tests (30-second sit-to-stand, timed up and go), and **aerobic testing (6-minute walk test)**
- Outcome Measures:
  - Edmonton Symptom Assessment Scale
  - Godin-Leisure Time Questionnaire
  - Functional Assessment of Cancer Therapy – General (FACT-G)
- **Note: The PT Navigator will use their clinical training and reasoning to personalize the assessment given each patient they see. The tests listed above are proposed as a standardized set of measures to use but the PT Navigator will not be limited to only those tests. For example, if an individual is living with breast cancer, the PT Navigator can add a lymphedema screening and relevant assessments to their follow up.**

Please rate the following statement: **The assessment is clear and easy to follow.**

Please choose the appropriate response for each item:

| **Strongly Disagree** | **Disagree** | **Somewhat Disagree** | **Neutral** | **Somewhat Agree** | **Agree** | **Strongly Agree** |
| --- | --- | --- | --- | --- | --- | --- |

If you rated neutral or below, please explain your selection provided above.

Please write your answer here:

Please rate the following statement: **I am satisfied with the assessment of the PT Navigator.**

Please choose the appropriate response for each item:

| **Strongly Disagree** | **Disagree** | **Somewhat Disagree** | **Neutral** | **Somewhat Agree** | **Agree** | **Strongly Agree** |
| --- | --- | --- | --- | --- | --- | --- |

If you rated neutral or below, please explain your selection provided above.

Please write your answer here:

*Triaging Decisions*

- The PT Navigator will move forward with one of the following steps based on their assessment:
  - 1) Deliver a physiotherapy intervention (i.e., provide education, exercise prescription, goal setting, rehabilitation barrier identification)
  - 2) Refer the individual to a community program (i.e., the patient needs more frequent follow-up and/or more supervision with rehabilitation services)
  - 3) Refer to another health care discipline (i.e., the patient needs care outside of the PT scope of practice – for example, to discuss changes in weight and appetite with a dietician)
  - 4) Continue with ongoing surveillance (i.e., no complications of chemotherapy treatment identified – continue to provide positive reassurance, answer questions, and provide education on maintenance strategies)
- Below is an accompanying guide created to support triaging based on outcome-assessment scores: *(*Please note that the changes to the chart are identified in BOLD in the Goniometry/Grip Strength, 30-Second Sit to Stand, Timed Up and Go, and 6-Minute Walk Test sections)*

Please rate the following statement: **The triaging decisions are clear and easy to follow.**

Please choose the appropriate response for each item:

| **Strongly Disagree** | **Disagree** | **Somewhat Disagree** | **Neutral** | **Somewhat Agree** | **Agree** | **Strongly Agree** |
| --- | --- | --- | --- | --- | --- | --- |

If you rated neutral or below, please explain your selection provided above.

Please write your answer here:

Please rate the following statement: **I am satisfied with the triaging decisions of the PT Navigator.**

Please choose the appropriate response for each item:

| **Strongly Disagree** | **Disagree** | **Somewhat Disagree** | **Neutral** | **Somewhat Agree** | **Agree** | **Strongly Agree** |
| --- | --- | --- | --- | --- | --- | --- |

If you rated neutral or below, please explain your selection provided above.

Please write your answer here:

*Duties and Responsibilities*

The following picture contains the main duties and responsibilities proposed for the PT Navigator. *(Note: No changes were made to this section as no responses received 10% agreement)*

Please rate the following statement: **The duties and responsibilities of the PT Navigator are clear and easy to follow.**

Please choose the appropriate response for each item:

If you rated neutral or below, please explain your selection provided above.

| **Strongly Disagree** | **Disagree** | **Somewhat Disagree** | **Neutral** | **Somewhat Agree** | **Agree** | **Strongly Agree** |
| --- | --- | --- | --- | --- | --- | --- |

Please write your answer here:

Please rate the following statement: **I am satisfied with the duties and responsibilities of the PT Navigator.**

Please choose the appropriate response for each item:

| **Strongly Disagree** | **Disagree** | **Somewhat Disagree** | **Neutral** | **Somewhat Agree** | **Agree** | **Strongly Agree** |
| --- | --- | --- | --- | --- | --- | --- |

If you rated neutral or below, please explain your selection provided above.

Please write your answer here:

*Figure*

Based on feedback from the first survey, we updated the figure to reduce the amount of text, colours, and decrease the number of arrows/change their directionality.

Below are two figures - the figure on the **LEFT** is from survey one, the figure on the **RIGHT** is the updated figure.

Please rate the following statement: **The updated figure is clear and easy to follow.** (reminder: the figure on the **RIGHT**)

Please choose the appropriate response for each item:

| **Strongly Disagree** | **Disagree** | **Somewhat Disagree** | **Neutral** | **Somewhat Agree** | **Agree** | **Strongly Agree** |
| --- | --- | --- | --- | --- | --- | --- |

If you rated neutral or below, please explain your selection provided above.

Please write your answer here:

Please rate the following statement: **I am satisfied with the updated figure.** (reminder: the figure on the **RIGHT**)

Please choose the appropriate response for each item:

| **Strongly Disagree** | **Disagree** | **Somewhat Disagree** | **Neutral** | **Somewhat Agree** | **Agree** | **Strongly Agree** |
| --- | --- | --- | --- | --- | --- | --- |

If you rated neutral or below, please explain your selection provided above.

Please write your answer here:

*Thank you*

Thank you so much for taking the time to complete the second survey! If you consented to receive the results in survey one, once results are finalized and ready to be presented you will receive an email with the final role description.

Thank you for taking this survey. Your answers are a valuable part of this research.
